# Supplementary figures and images for: Inhibiting NINJ1-dependent plasma membrane rupture protects against inflammasome-induced blood coagulation and inflammation
Source: eLife. 2025 Mar 17;12:RP91329. doi: 10.7554/eLife.91329 (PMC11913443; doi:10.7554/eLife.91329)

Figure 1G–source data

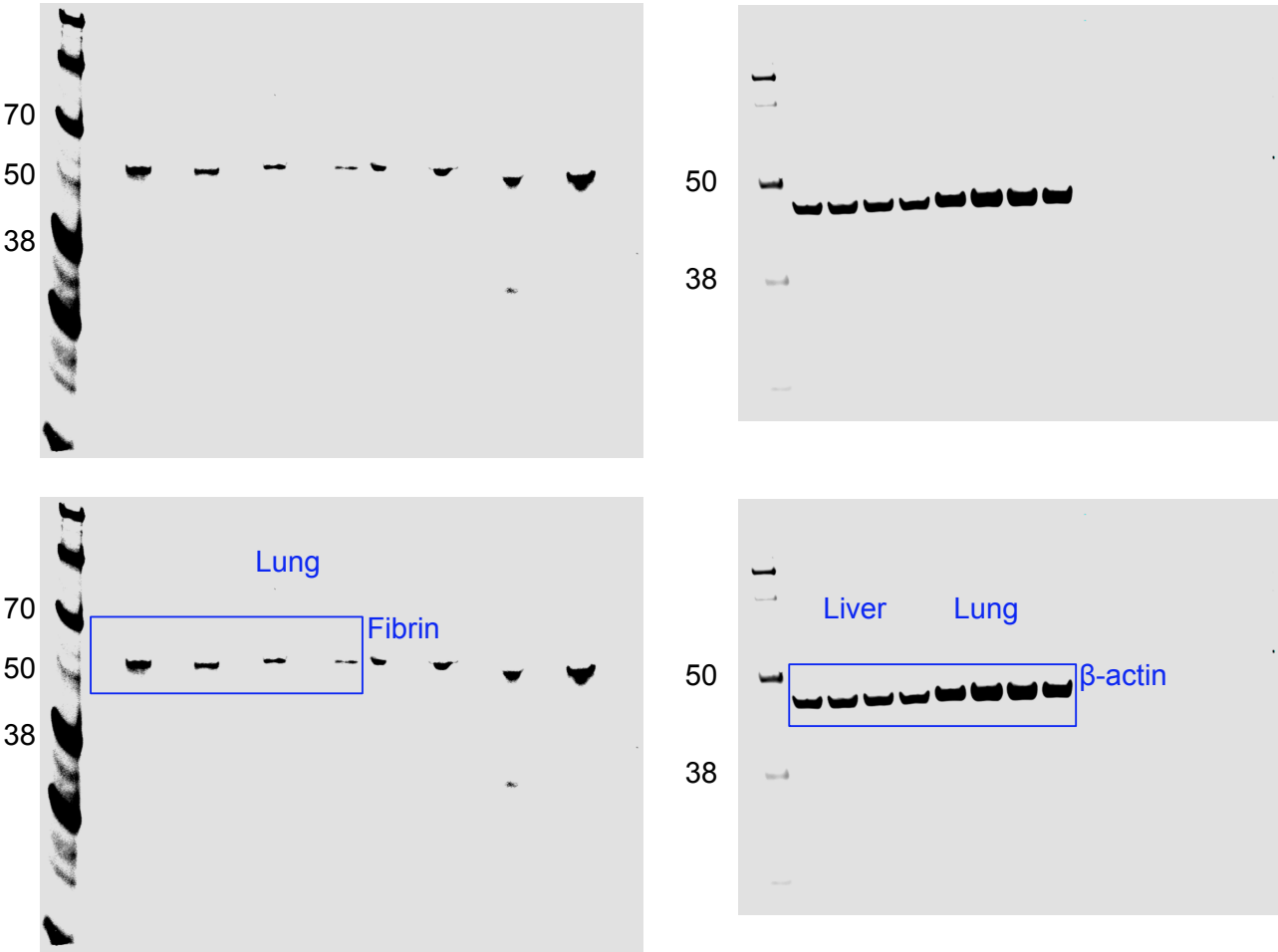

Supplement: Figure 1—source data 2. [file elife-91329-fig1-data2.zip › Figure 1-source data 2.pdf]

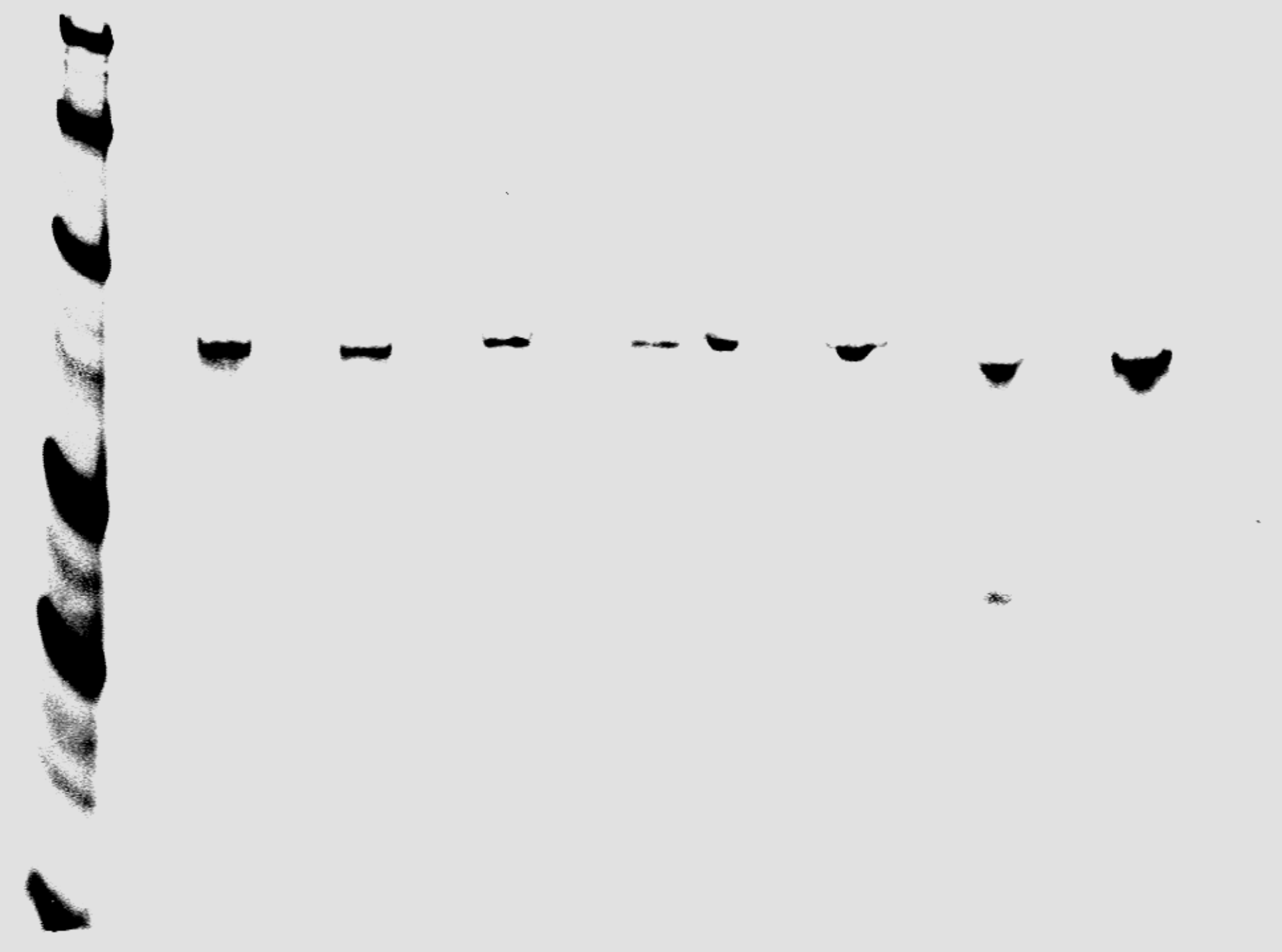

Supplement: Figure 1—source data 3. [file elife-91329-fig1-data3.zip › Figure 1-source data 3/Figure-1G-Fibrin.tif]

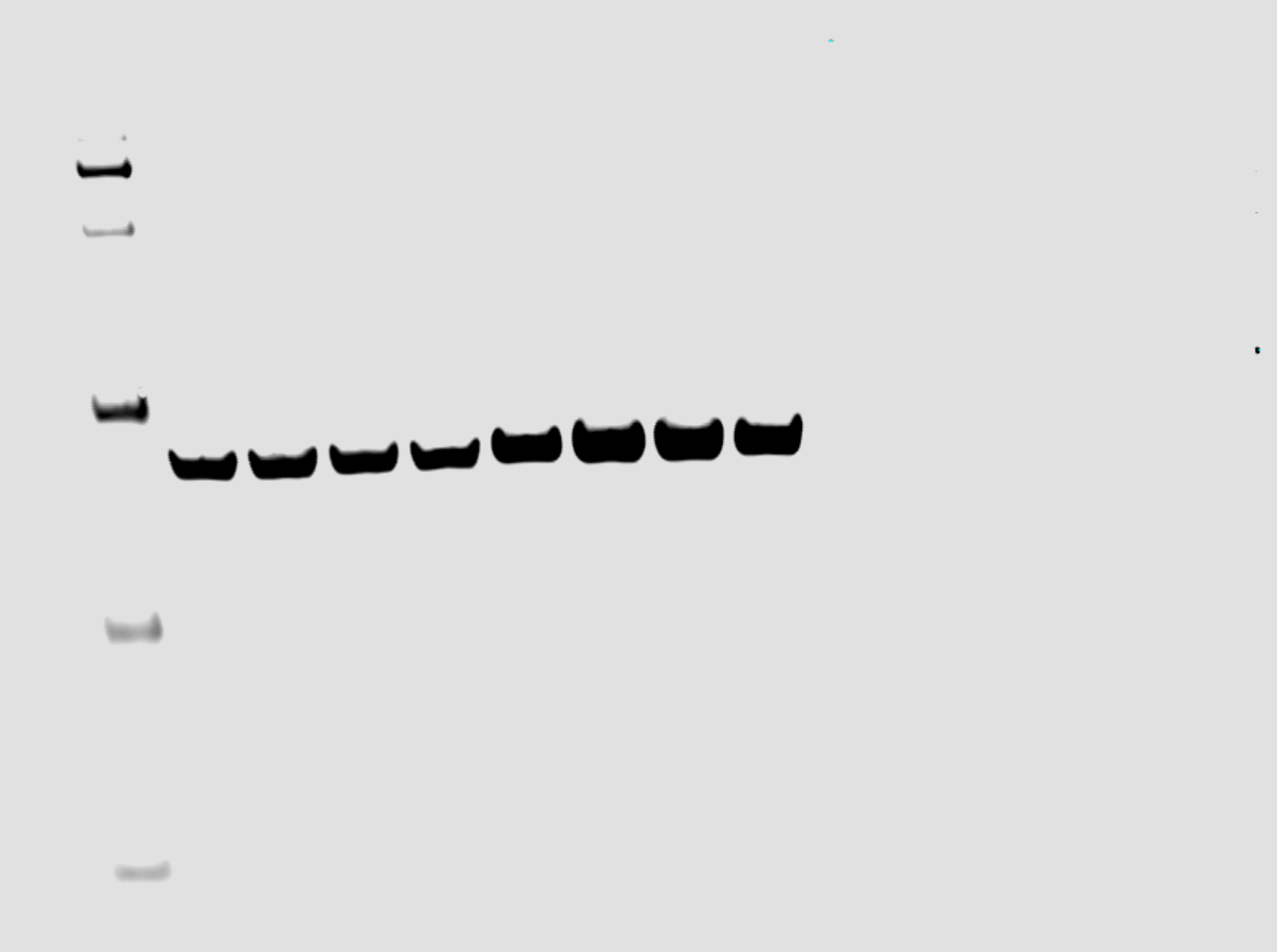

Supplement: Figure 1—source data 3. [file elife-91329-fig1-data3.zip › Figure 1-source data 3/Figure-1G-Actin.tif]

Figure 1–figure supplement 1–source data

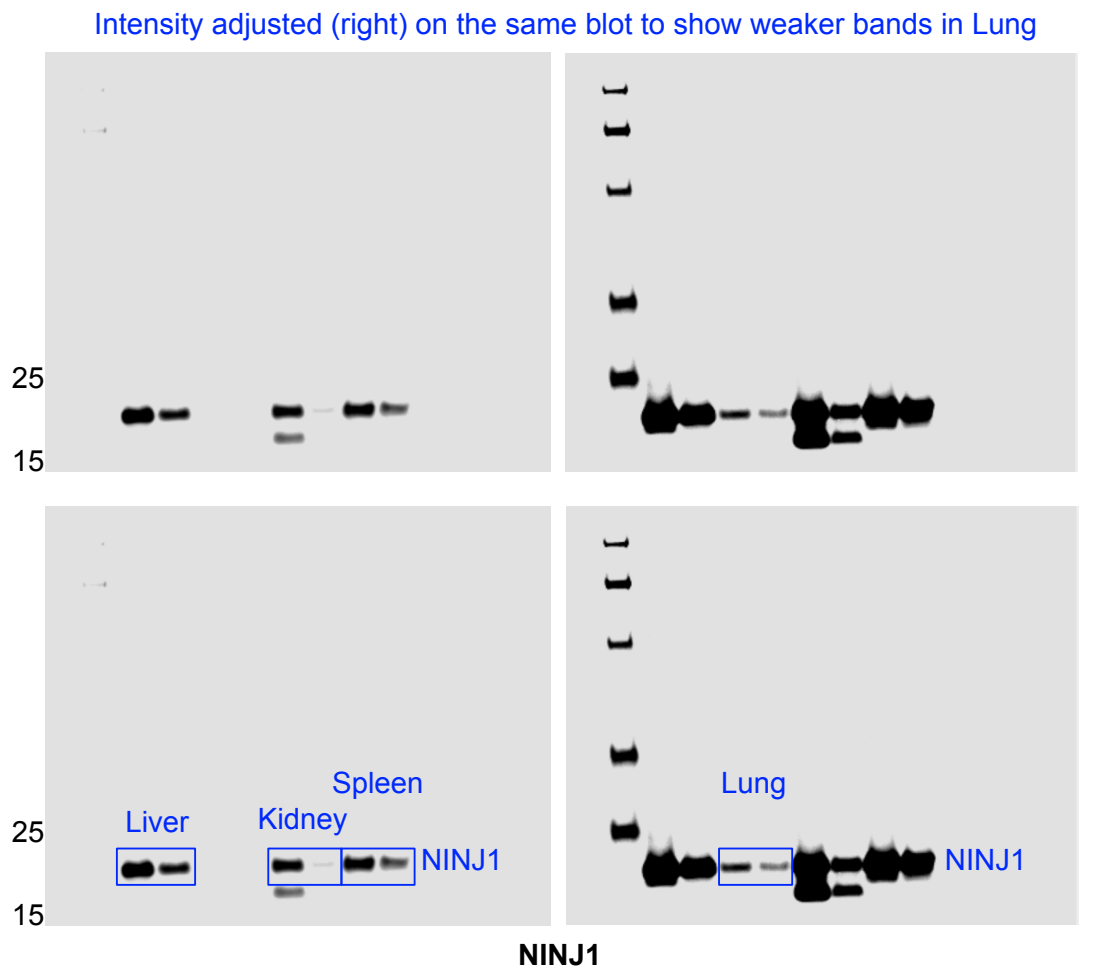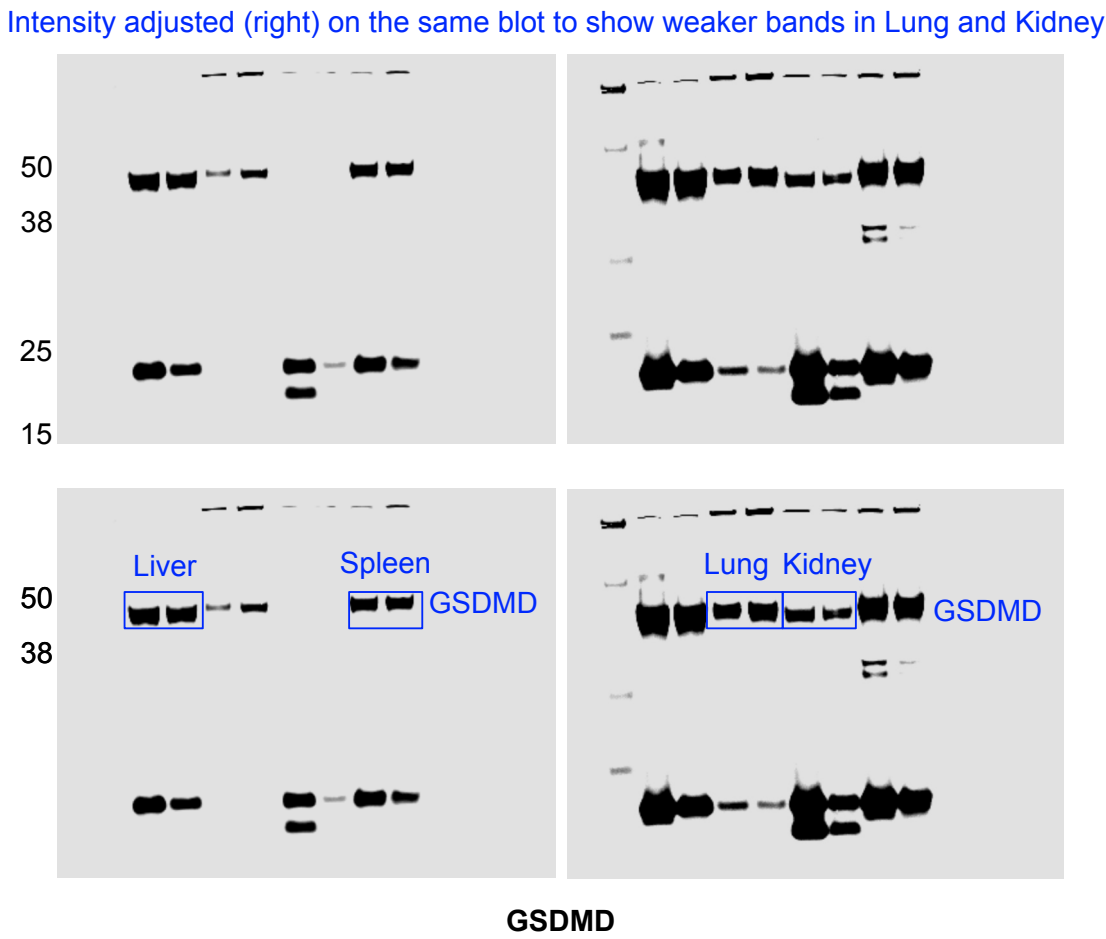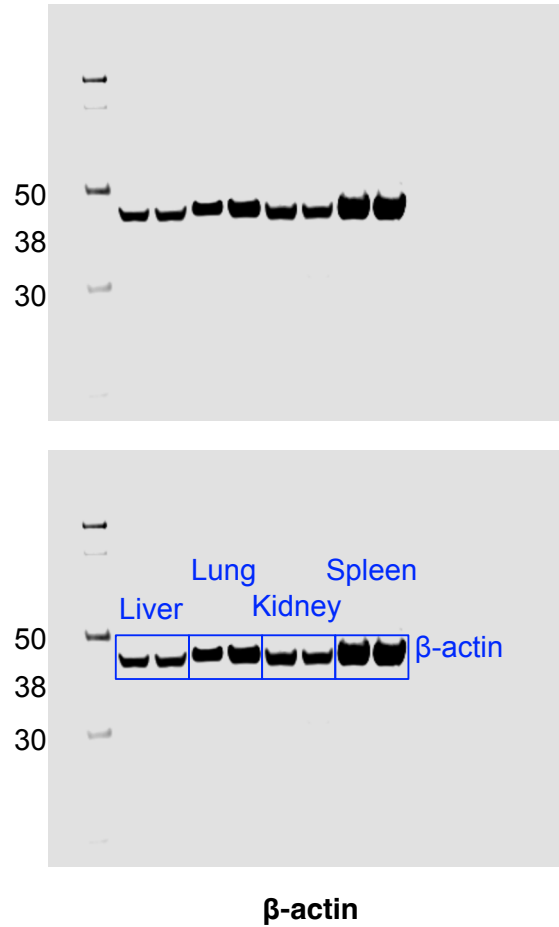

Supplement: Figure 1—figure supplement 1—source data 1. [file elife-91329-fig1-figsupp1-data1.zip › Figure 1-figure supplement 1-Source data 1.pdf]

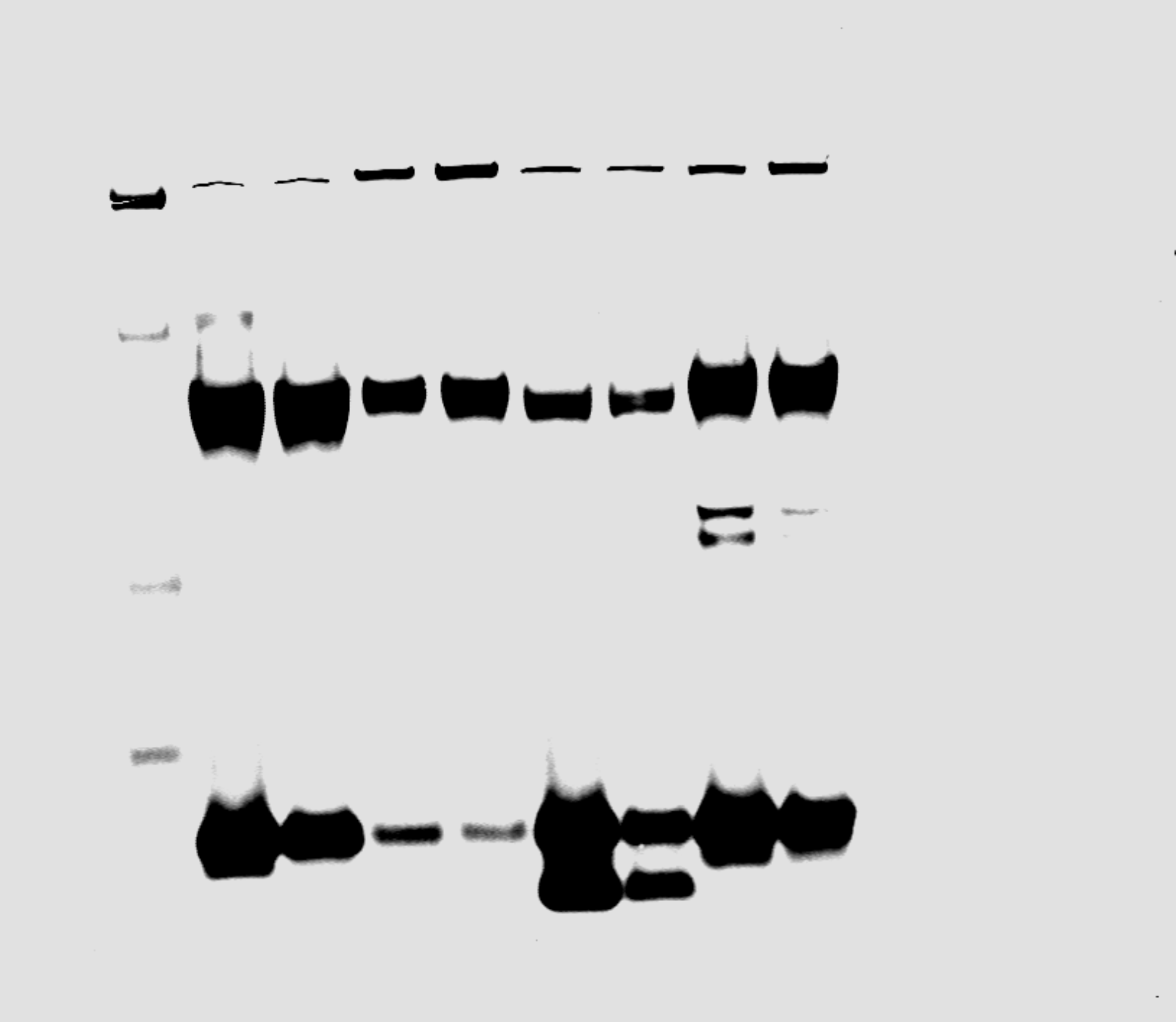

Supplement: Figure 1—figure supplement 1—source data 2. [file elife-91329-fig1-figsupp1-data2.zip › Figure 1-figure supplement 1-Source data 2/Figure 1-figure supplement 1-GSDMD.tif]

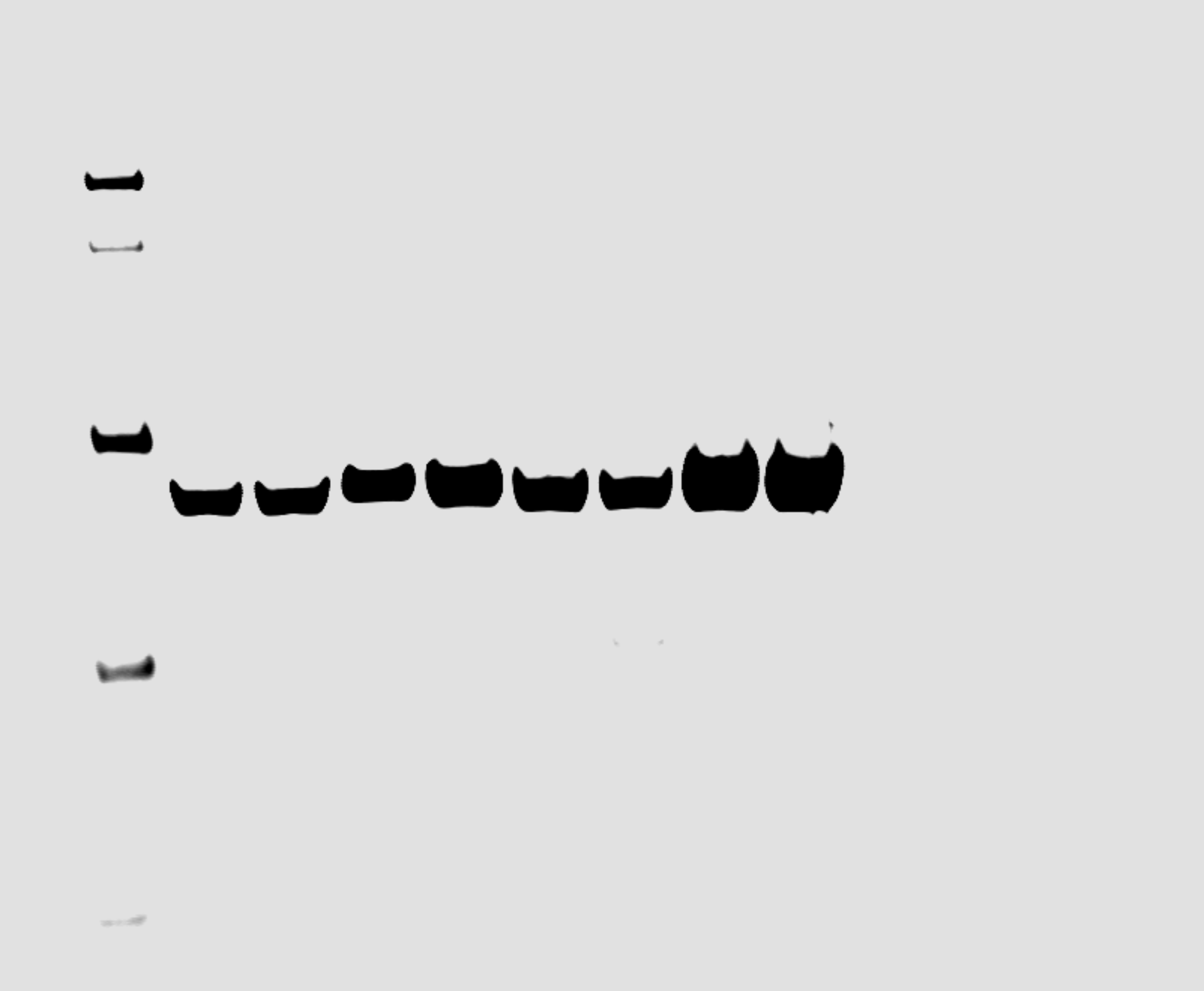

Supplement: Figure 1—figure supplement 1—source data 2. [file elife-91329-fig1-figsupp1-data2.zip › Figure 1-figure supplement 1-Source data 2/Figure 1-figure supplement 1-Actin.tif]

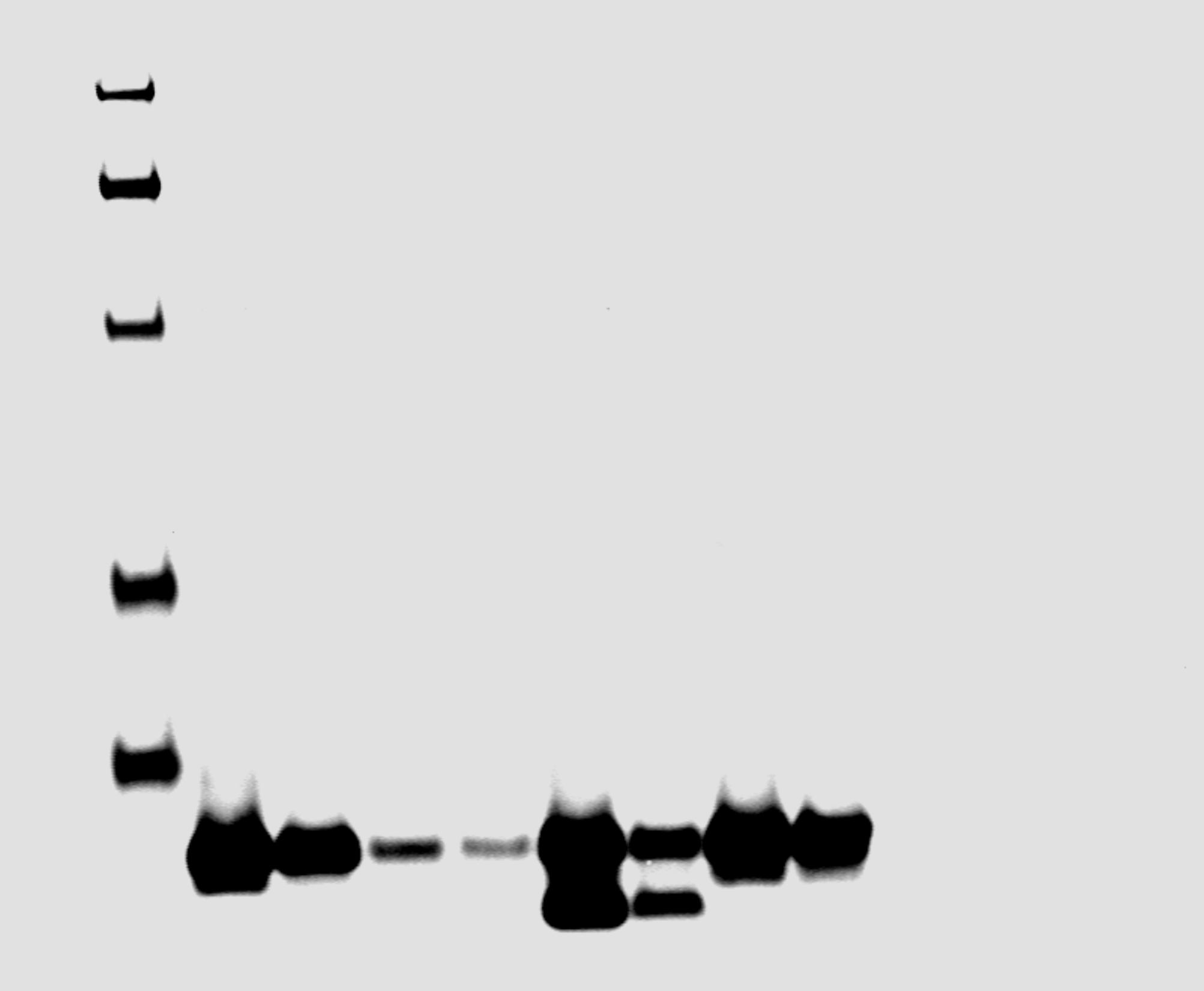

Supplement: Figure 1—figure supplement 1—source data 2. [file elife-91329-fig1-figsupp1-data2.zip › Figure 1-figure supplement 1-Source data 2/Figure 1-figure supplement 1-NINJ1.tif]

Figure 1–figure supplement 2B–source data

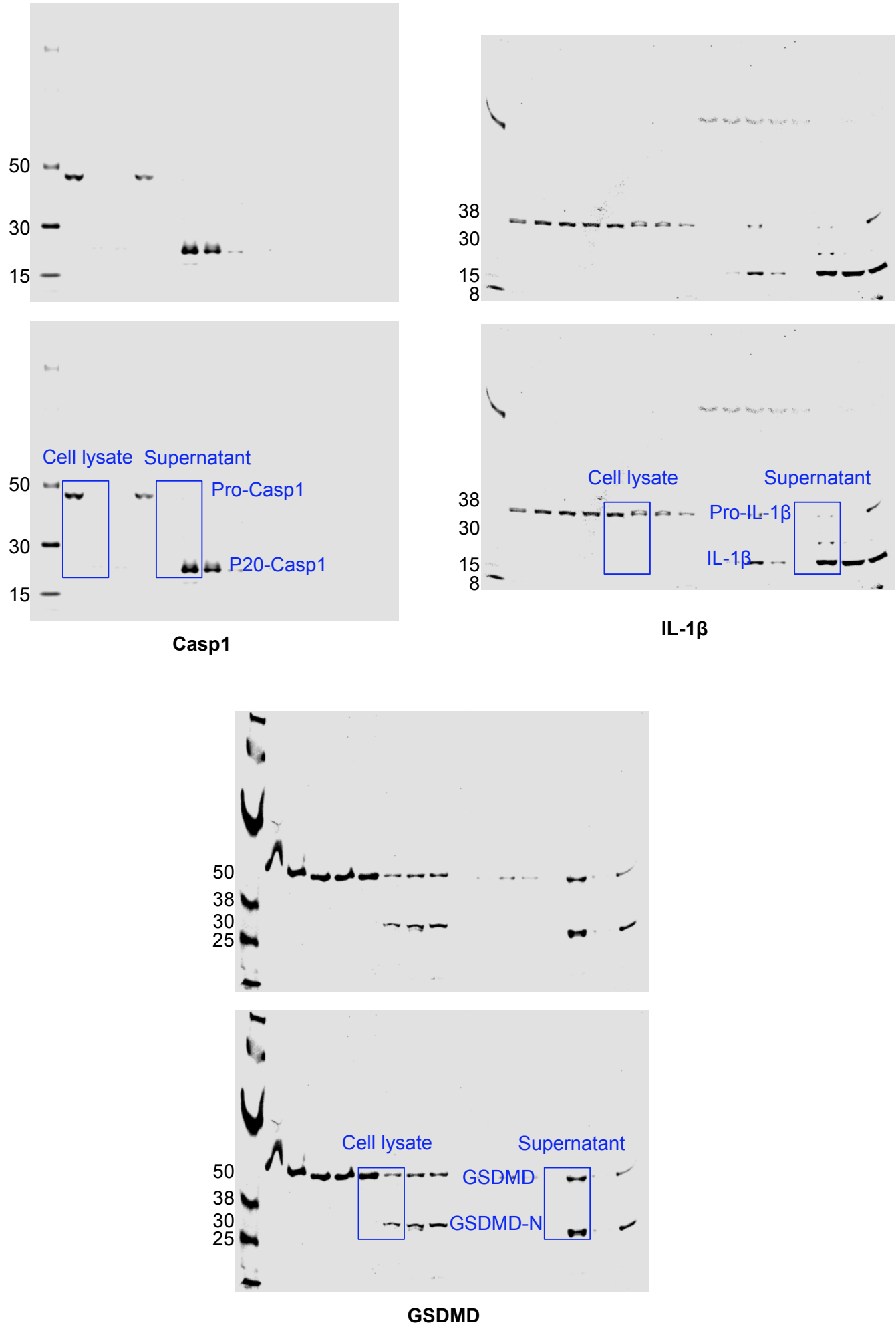

Supplement: Figure 1—figure supplement 2—source data 2. [file elife-91329-fig1-figsupp2-data2.zip › Figure 1-figure supplement 2-Source data 2.pdf]

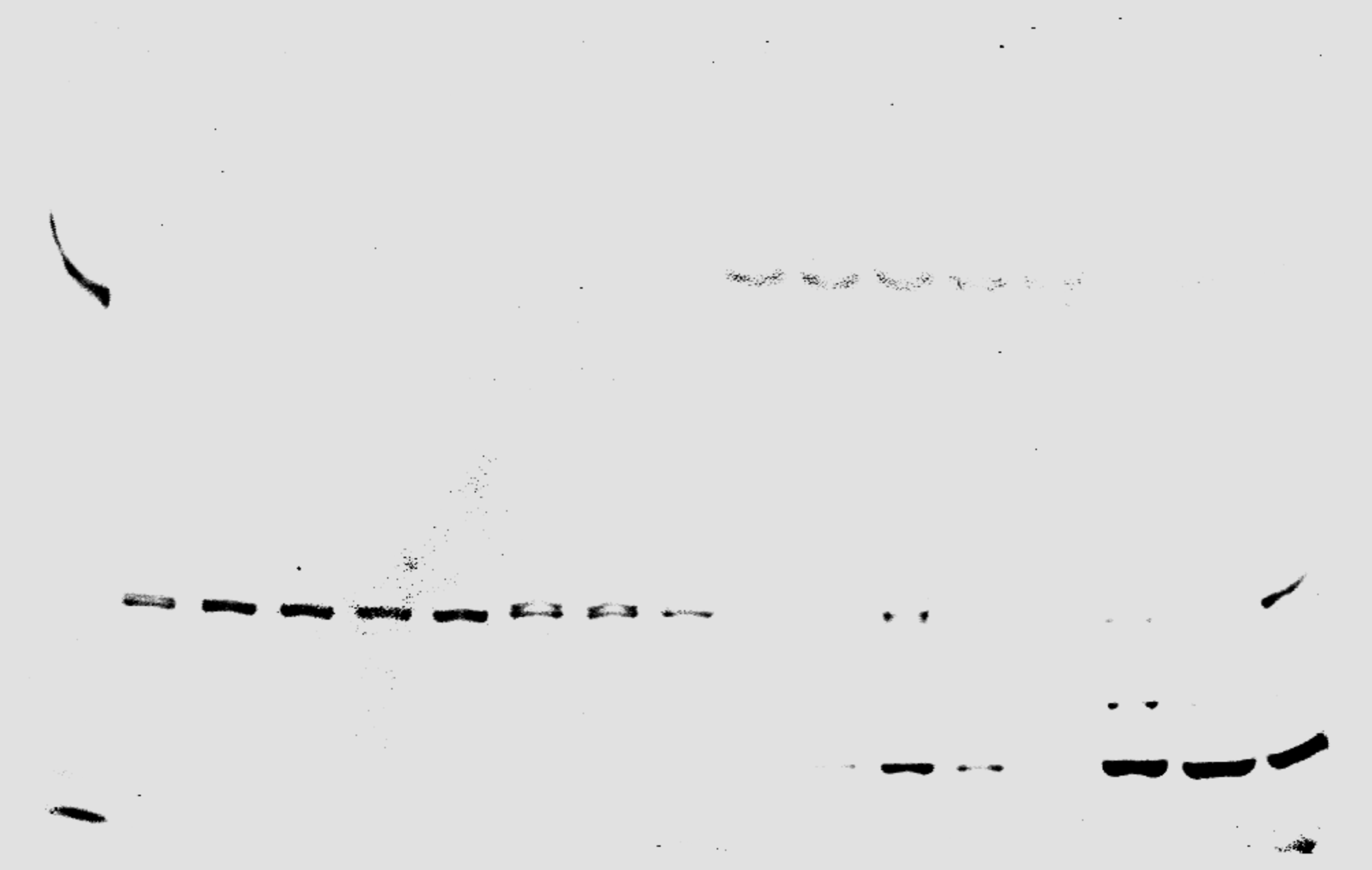

Supplement: Figure 1—figure supplement 2—source data 3. [file elife-91329-fig1-figsupp2-data3.zip › Figure 1-figure supplement 2-Source data 3/Figure 1-figure supplement 2B-IL-1b.tif]

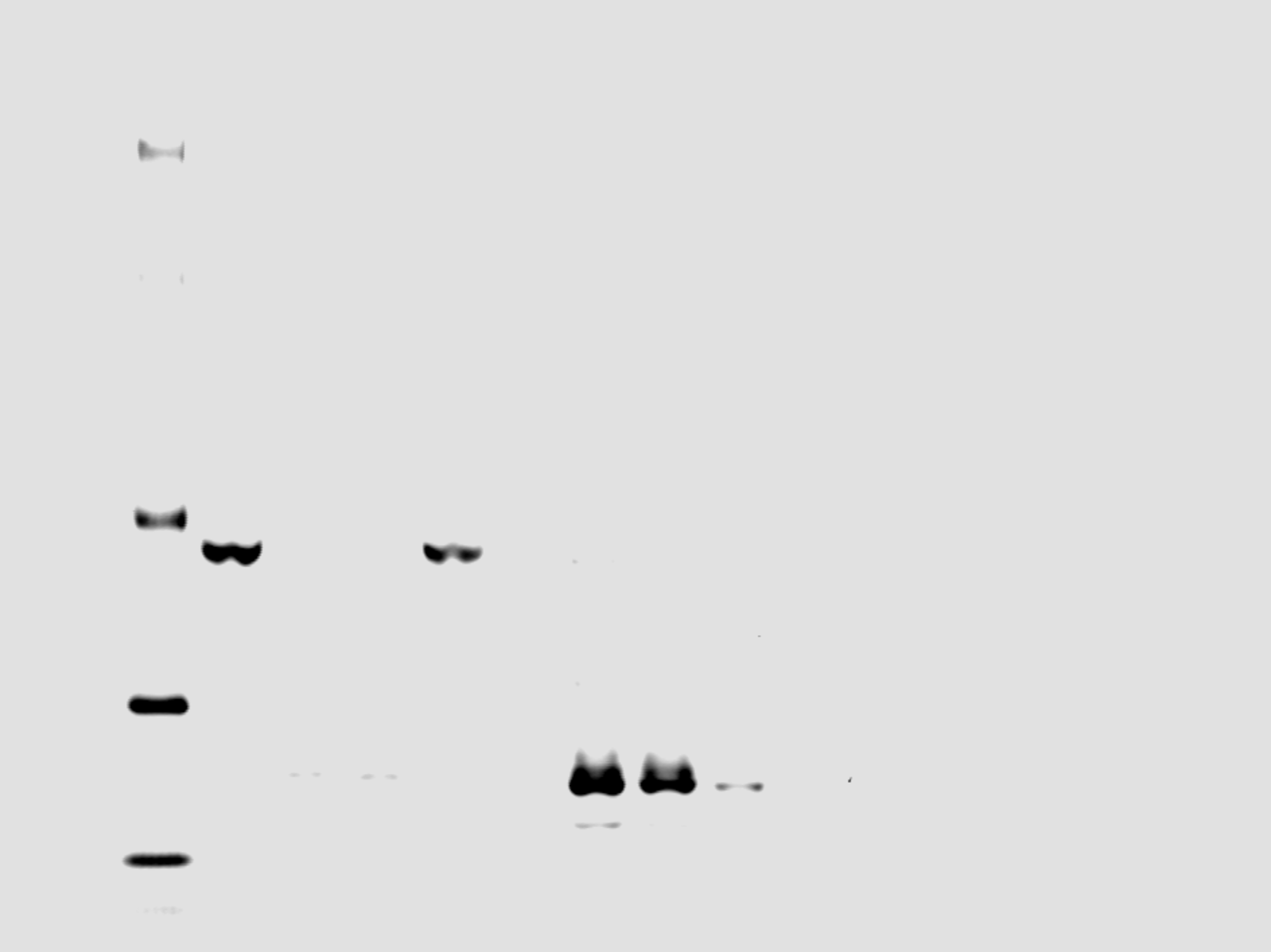

Supplement: Figure 1—figure supplement 2—source data 3. [file elife-91329-fig1-figsupp2-data3.zip › Figure 1-figure supplement 2-Source data 3/Figure 1-figure supplement 2B-Casp1.tif]

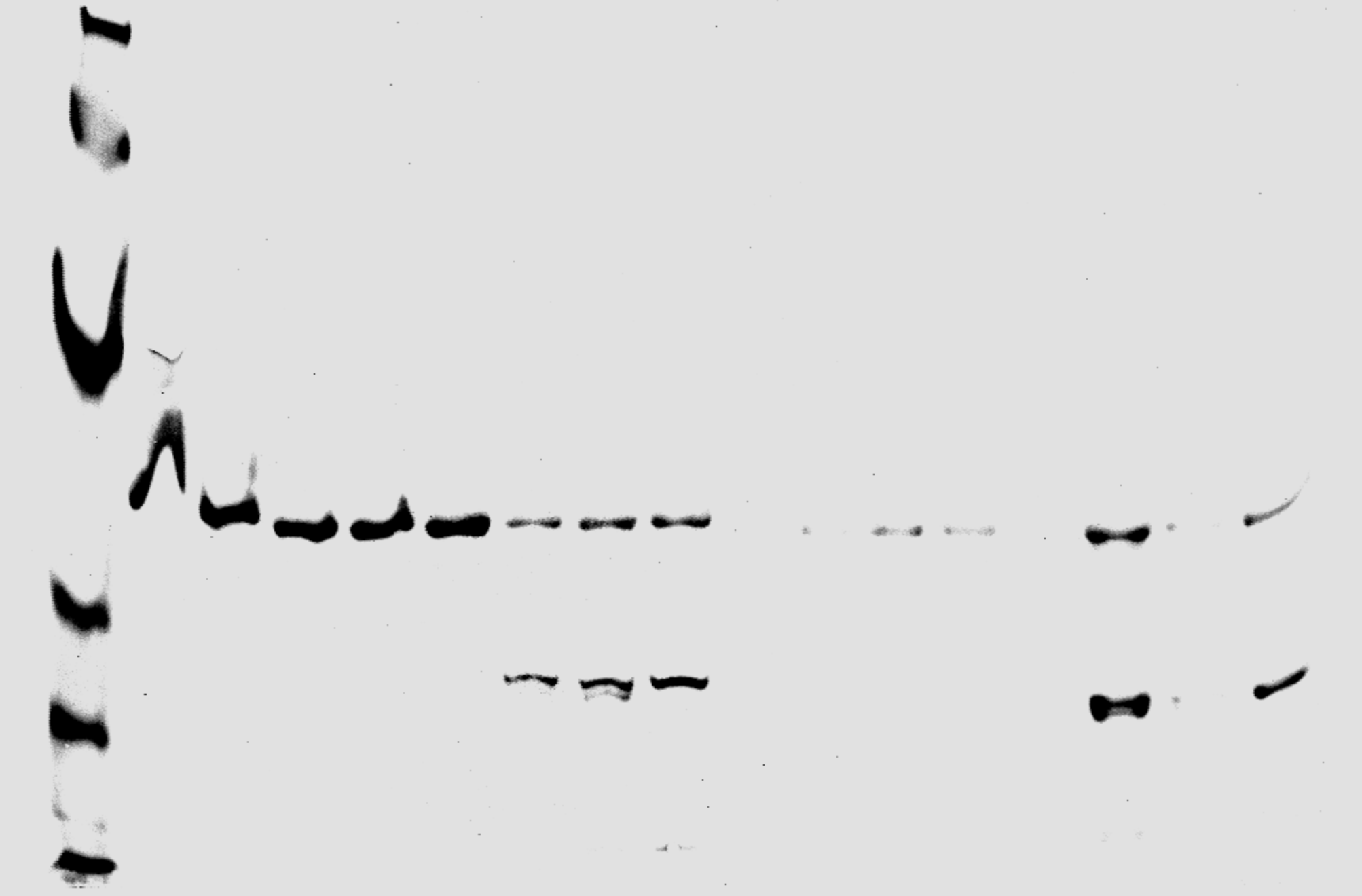

Supplement: Figure 1—figure supplement 2—source data 3. [file elife-91329-fig1-figsupp2-data3.zip › Figure 1-figure supplement 2-Source data 3/Figure 1-figure supplement 2B-GSDMD.tif]

Figure 3A–source data

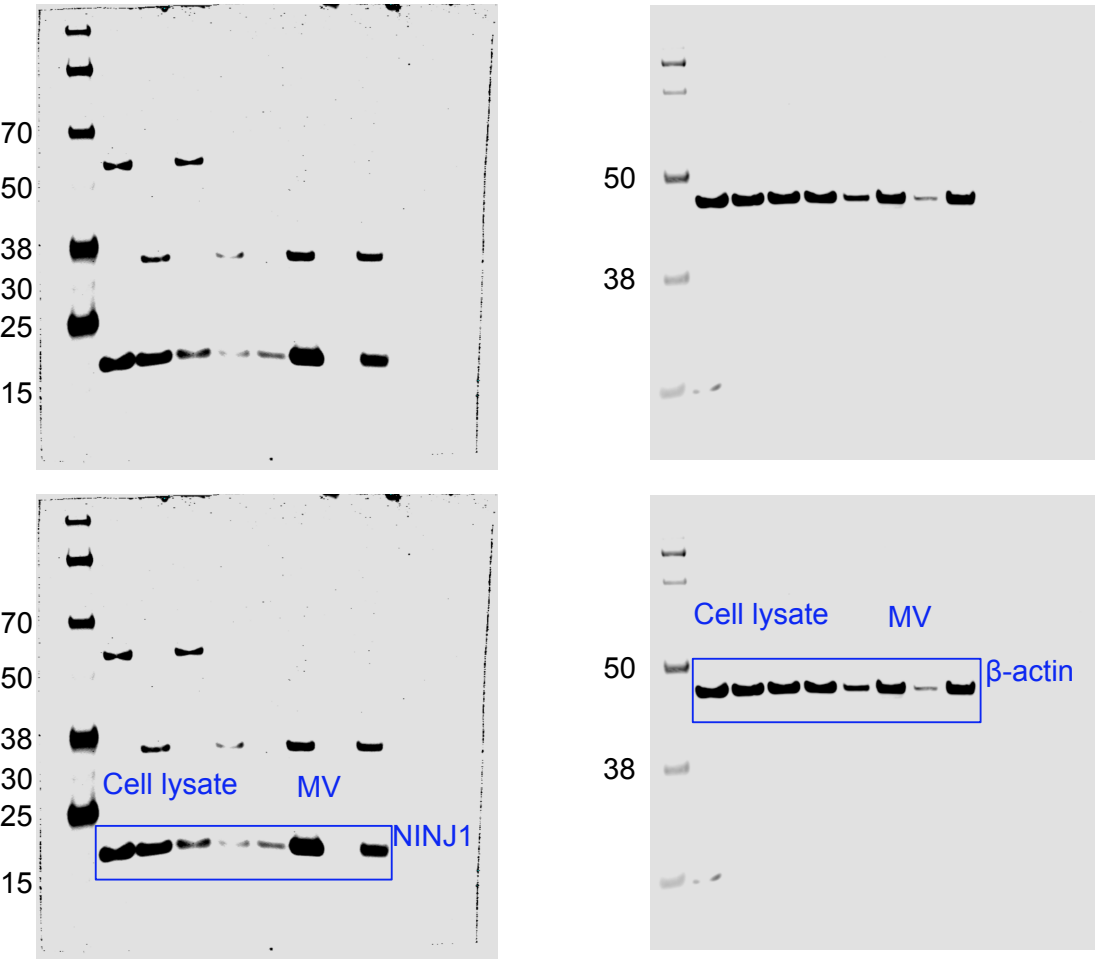

Figure 3D–source data

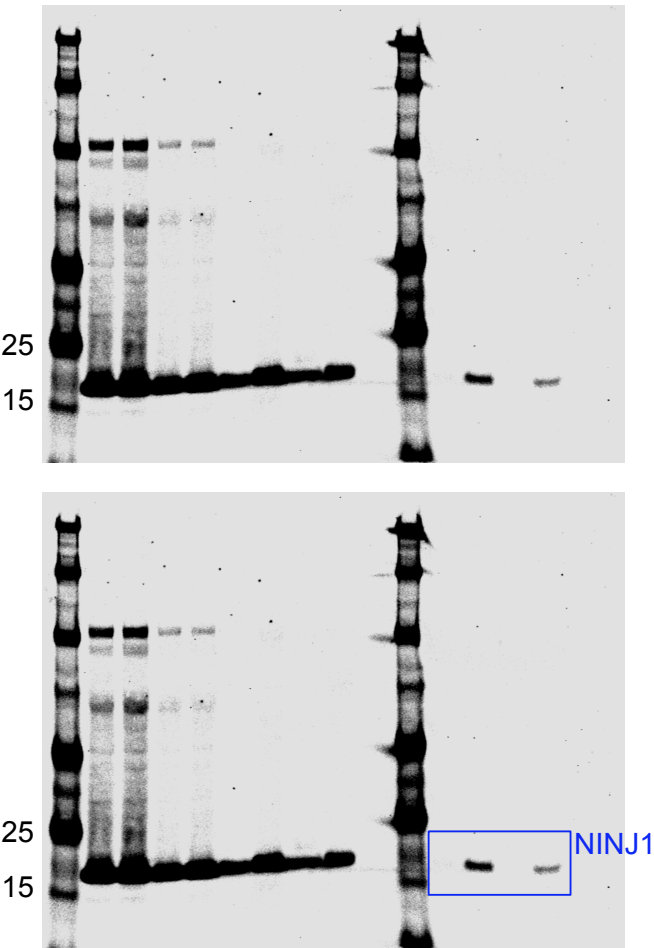

Supplement: Figure 3—source data 1. [file elife-91329-fig3-data1.zip › Figure 3-Source data 1.pdf]

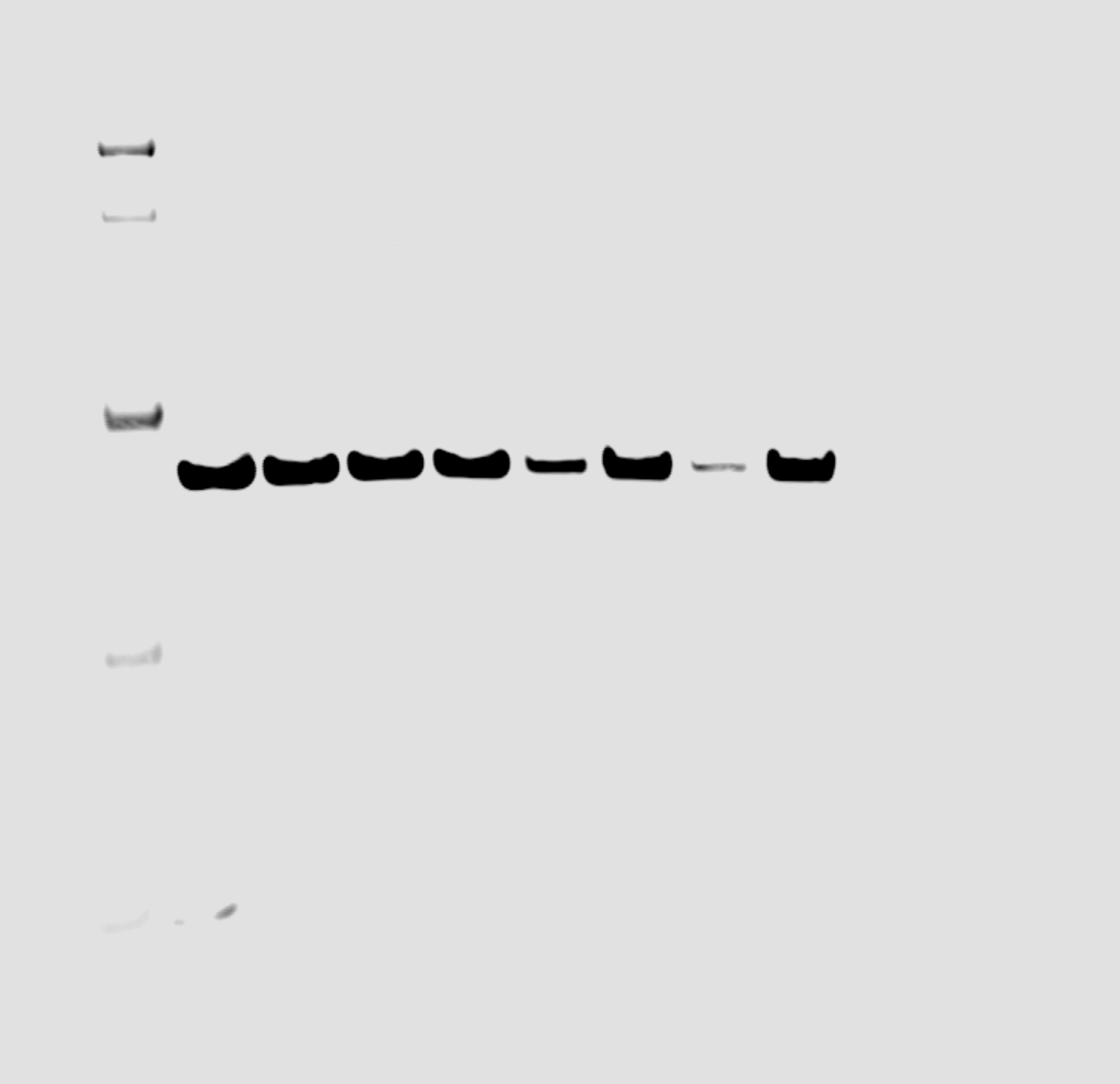

Supplement: Figure 3—source data 2. [file elife-91329-fig3-data2.zip › Figure 3-Source data 2/Figure 3A-Actin.tif]

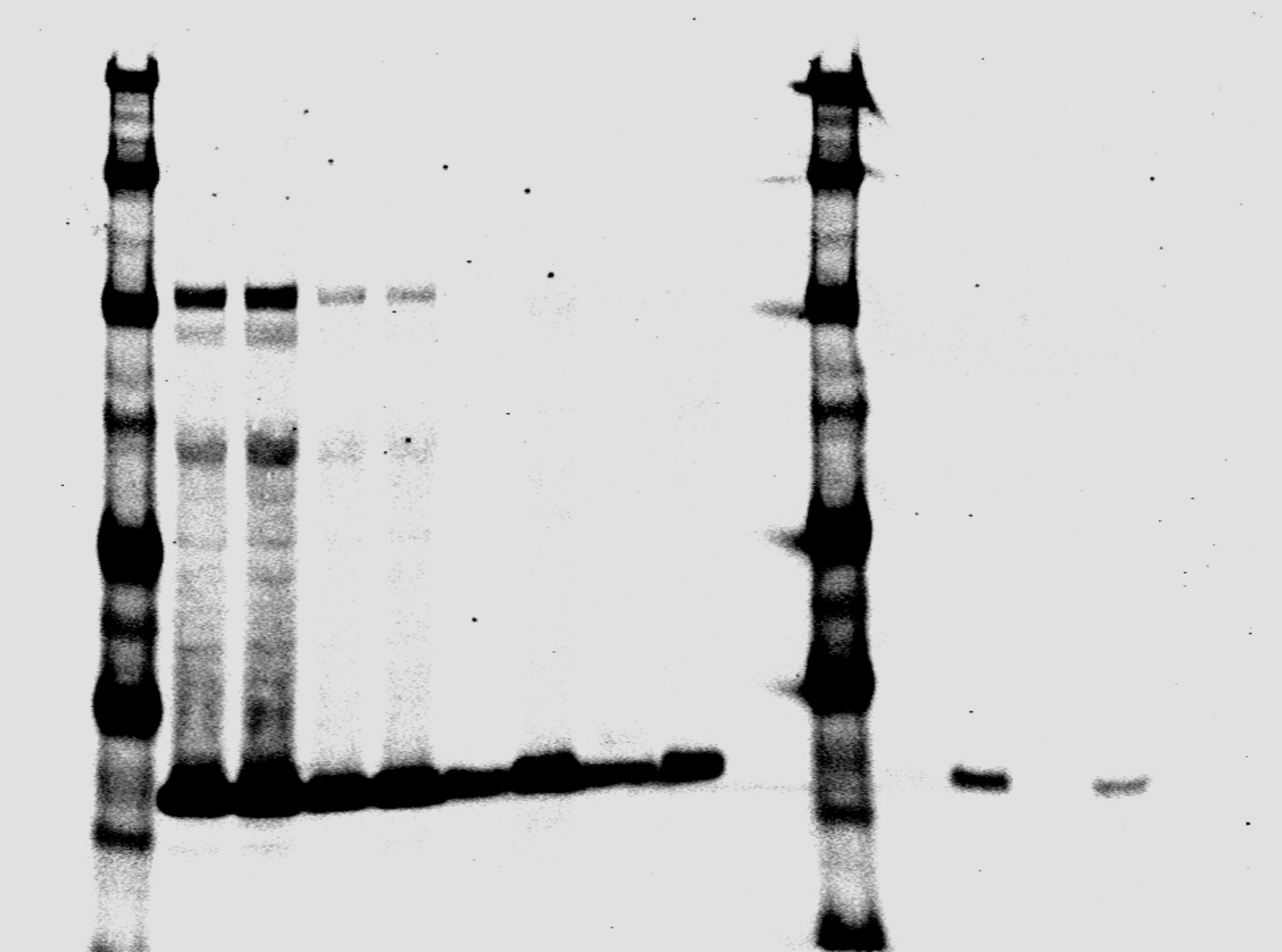

Supplement: Figure 3—source data 2. [file elife-91329-fig3-data2.zip › Figure 3-Source data 2/Figure 3D-NINJ1.tif]

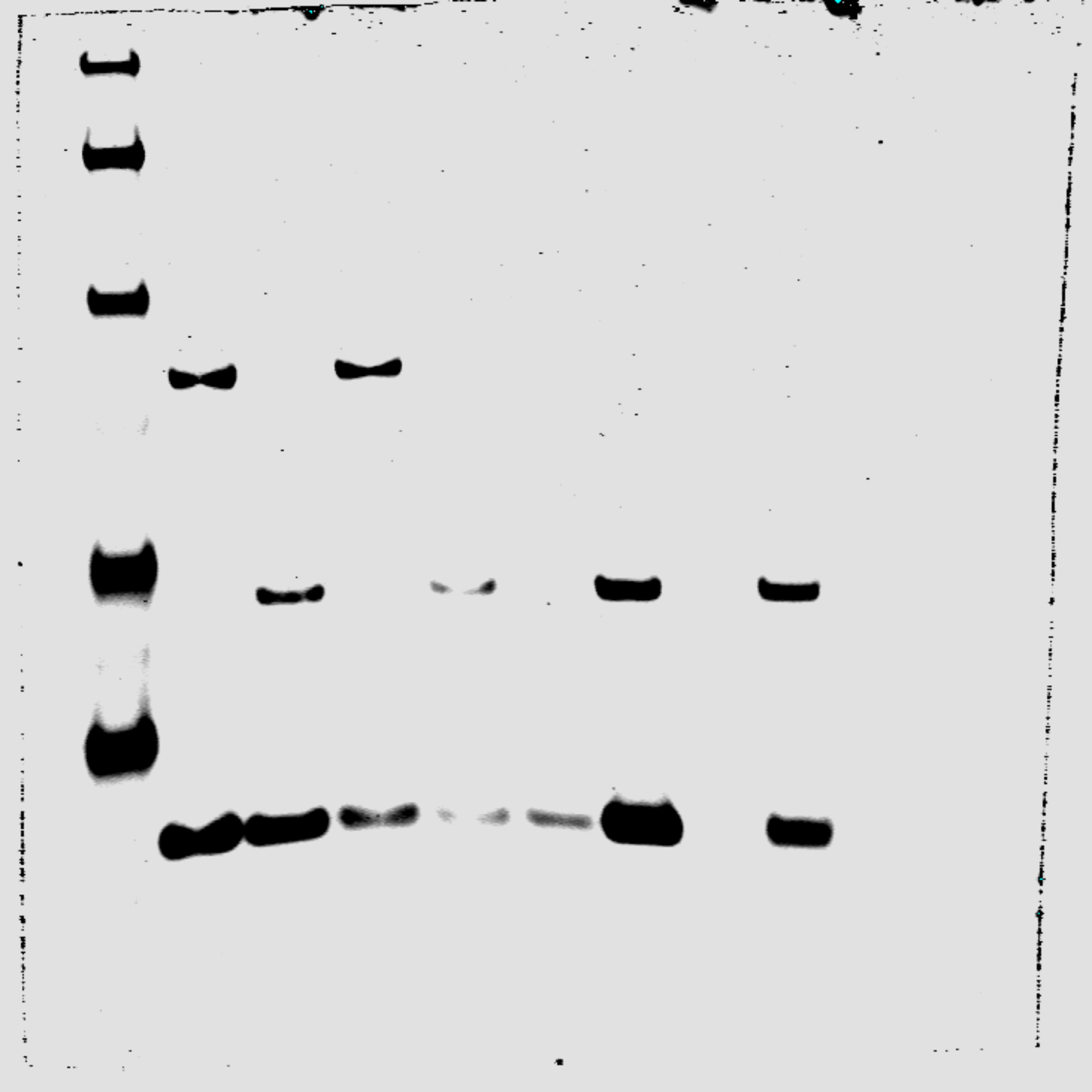

Supplement: Figure 3—source data 2. [file elife-91329-fig3-data2.zip › Figure 3-Source data 2/Figure 3A-NINJ1.tif]
